# Enhancing the Resolution of Rumen Microbial Classification from Metatranscriptomic Data Using Kraken and Mothur

**DOI:** 10.3389/fmicb.2017.02445

**Published:** 2017-12-07

**Authors:** Andre L. A. Neves, Fuyong Li, Bibaswan Ghoshal, Tim McAllister, Le L. Guan

**Affiliations:** ^1^Department of Agricultural, Food and Nutritional Science, University of Alberta, Edmonton, AB, Canada; ^2^Lethbridge Research Centre, Agriculture and Agri-Food Canada, Lethbridge, AB, Canada

**Keywords:** rumen microbiota, bacteria, archaea, kraken, mothur

## Abstract

The advent of next generation sequencing and bioinformatics tools have greatly advanced our knowledge about the phylogenetic diversity and ecological role of microbes inhabiting the mammalian gut. However, there is a lack of information on the evaluation of these computational tools in the context of the rumen microbiome as these programs have mostly been benchmarked on real or simulated datasets generated from human studies. In this study, we compared the outcomes of two methods, Kraken (mRNA based) and a pipeline developed in-house based on Mothur (16S rRNA based), to assess the taxonomic profiles (bacteria and archaea) of rumen microbial communities using total RNA sequencing of rumen fluid collected from 12 cattle with differing feed conversion ratios (FCR). Both approaches revealed a similar phyla distribution of the most abundant taxa, with Bacteroidetes, Firmicutes, and Proteobacteria accounting for approximately 80% of total bacterial abundance. For bacterial taxa, although 69 genera were commonly detected by both methods, an additional 159 genera were exclusively identified by Kraken. Kraken detected 423 species, while Mothur was not able to assign bacterial sequences to the species level. For archaea, both methods generated similar results only for the abundance of Methanomassiliicoccaceae (previously referred as RCC), which comprised more than 65% of the total archaeal families. Taxon R4-41B was exclusively identified by Mothur in the rumen of feed efficient bulls, whereas Kraken uniquely identified Methanococcaceae in inefficient bulls. Although Kraken enhanced the microbial classification at the species level, identification of bacteria or archaea in the rumen is limited due to a lack of reference genomes for the rumen microbiome. The findings from this study suggest that the development of the combined pipelines using Mothur and Kraken is needed for a more inclusive and representative classification of microbiomes.

## Introduction

The success of microbiome studies (composition, structure, diversity, and function) is primarily ascribable to the development of bioinformatics tools embedded in creative algorithms specially tailored to overcome the technical challenges posed by the analysis of massively paralleled, high-throughput sequencing data (Simon and Daniel, 2011; Siegwald et al., 2017). These bioinformatics tools make use of several techniques (e.g., read mapping, k-mer alignment, and composition analysis) (Piro et al., 2017) and can be categorized into two distinct groups: (1) programs that use all available genome sequences (Lindgreen et al., 2016), also called assignment-first approaches (Siegwald et al., 2017) (e.g., CLARK – Ounit et al., 2015; GOTTCHA – Freitas et al., 2015; KRAKEN – Wood and Salzberg, 2014; MG-RAST – Meyer et al., 2008), and (2) programs that target a set of marker genes (Lindgreen et al., 2016), also known as clustering-first approaches (Siegwald et al., 2017) (e.g., QIIME – Caporaso et al., 2010; MOTHUR – Schloss et al., 2009; MetaPhlAn – Segata et al., 2012; mOTU – Sunagawa et al., 2013). In the assignment-first tools, all reads are assigned to the lowest taxonomy unit (lower common ancestor-LCA) within a reference database based on their annotations, while in the clustering-first approaches the reads are grouped into Operational Taxonomic Units (OTUs) using different OTU picking strategies (closed or open reference) to assign reads to a taxonomic group based on their sequence similarities (Siegwald et al., 2017).

However, most of the above studies are focused on demonstrating how single analytical steps (e.g., sequence pre-processing, OTU clustering or taxonomic assignment) generated by the existing tools impact the microbial classification in real or simulated datasets derived from the Human Microbiome Project (Siegwald et al., 2017). Comparison of methodologies to comprehensively classify the rumen microbiome is lacking which may be in part due to its complexity, as the rumen microbial community consists of bacteria, archaea, protozoa and fungi (Russell and Rychlik, 2001). A recent study by Li F. et al. (2016) developed a Mothur (Schloss et al., 2009) based pipeline to assess active rumen microbiota from data generated from total RNA sequencing. Later, the same researchers applied this pipeline to investigate linkages between the active rumen microbiome (structure and function) and feed efficiency in beef cattle using metatranscriptomics (Li and Guan, 2017). Using the developed mothur-based pipeline for taxonomic assignment, the authors identified that the active microbial taxa differed in the rumen of cattle with differing feed efficiency and suggested that the active rumen microbiome is one of the biological factors that may contribute to variations in feed efficiency in beef cattle (Li and Guan, 2017). There were two steps employed in taxonomic classification by Li F. et al. (2016): bacterial sequences belonging to V1–V3 regions were extracted from the aligned Greengenes database, and archaeal sequences belonging to the V6–V8 regions were aligned with a rumen-specific archaeal 16S rRNA gene database (Janssen and Kirs, 2008). Despite the efficacy of this pipeline, it still remains a challenge for researchers to determine which approach (assignment- or clustering-first methods) of taxonomic classification delivers the most realistic representation of rumen microbial ecology.

In the current study, we propose a comparative analysis of the outcomes of Kraken (Wood and Salzberg, 2014) and the pipeline of Li F. et al. (2016) with a focus on the biological interpretation of the rumen microbial classification from the perspective of two conceptually different software packages. Unlike the pipeline developed by Li F. et al. (2016), Kraken algorithms can make multiple comparisons of single or assembled k-mers against any hypervariable region, providing useful information regarding a particular species detected in a region of the 16S rRNA gene that is different from the targeted internal conserved region initially sequenced (Wood and Salzberg, 2014; Valenzuela-González et al., 2016). Although Kraken algorithms have been originally designed to assign taxonomic identity to short DNA reads (Wood and Salzberg, 2014), studies have shown that Kraken is also useful to provide taxonomic classification for long (up to 1352.1 ± 153.72 bp) metagenomic DNA sequences (Valenzuela-González et al., 2016). Therefore, our objectives were (i) to compare and contrast the pipeline of Li F. et al. (2016) and Kraken to assess the taxonomic profiles of rumen bacteria and archaea and (ii) to investigate the impact of the comparative analysis of both analytical approaches on the biological interpretation of the rumen microbial classification obtained from cattle exhibiting different feed efficiencies.

## Materials and Methods

### Animal Study and Sampling

The experimental procedures described in this study were approved by the Veterinary Services and the Animal Care Committee, University of Manitoba, Canada, to ensure that animals were cared for in compliance with those ethics. Rumen contents were collected from 12 purebred Angus bulls (mean age of 249 ± 22 days and average body weight of 313.9 ± 32 kg) raised in confinement at the Glenlea Research Station located at the University of Manitoba according to the guidelines of the Canadian Council on Animal Care (CCAC) (Olfert et al., 1993), with bulls being fed a forage diet over two 80-day feeding periods (with a 20-day adaptation in between) as described by Thompson (2015). In the current study, 250 ml of rumen contents (liquid and solid fractions) were collected at the end of the second feeding period using a Geishauser oral probe (Duffield et al., 2004), immediately snap frozen in liquid nitrogen, and stored at -80°C for later processing. The feed intake of individual bulls was recorded using the GrowSafe^®^ feeding system (GrowSafe Systems Ltd., Airdrie, AB, Canada) and the feed conversion rate (FCR) was calculated as a ratio of dry matter intake to average daily gain (computed on a biweekly basis; Montanholi et al., 2010). The bulls were ranked into two groups: high (*n* = 6) and low (*n* = 6) FCR, with high (H-FCR) and low (L-FCR) standing for inefficient and efficient cattle in terms of diet utilization, respectively.

### RNA Extraction and Sequencing

Total RNA was extracted from rumen samples using the TRIzol protocol based on the acid guanidinium-phenol-chloroform method (Chomczynski and Sacchi, 2006; Béra-Maillet et al., 2009) with the modified procedures described by Li F. et al. (2016). Briefly, ∼200 mg of rumen sample was subjected to RNA extraction with the addition of 1.5 ml of TRIzol reagent (Invitrogen, Carlsbad, CA, United States), followed by 0.4 ml of chloroform, 0.3 ml of isopropanol, and 0.3 ml of high salt solution (1.2 M sodium acetate, 0.8 M NaCl) for the extraction protocol (Li F. et al., 2016). The yield and integrity of the RNA samples were determined using a Qubit 2.0 fluorimeter (Invitrogen, Carlsbad, CA, United States) and Agilent 2100 Bioanalyzer (Agilent Technologies, Santa Clara, CA, United States). RNA samples were subjected to downstream RNA-sequencing only if they exhibited RNA with integrity number (RIN) higher than 7.0. Briefly, total RNA (100 ng) of each sample was used for library construction using the TruSeq RNA sample prep v2 LS kit (Illumina, San Diego, CA, United States) without the mRNA enrichment step (Li F. et al., 2016). The quality of libraries was assessed using Agilent 2200 TapeStation (Agilent Technologies) and Qubit 2.0 fluorimeter (Invitrogen). Finally, cDNA fragments (∼140 bp) were paired-end (2 × 100 bp) sequenced using an Illumina HiSeq 2000 system at the McGill University and Génome Québec Innovation Centre (Montréal, QC, Canada).

### Pipeline Settings

**FIGURE 1:**
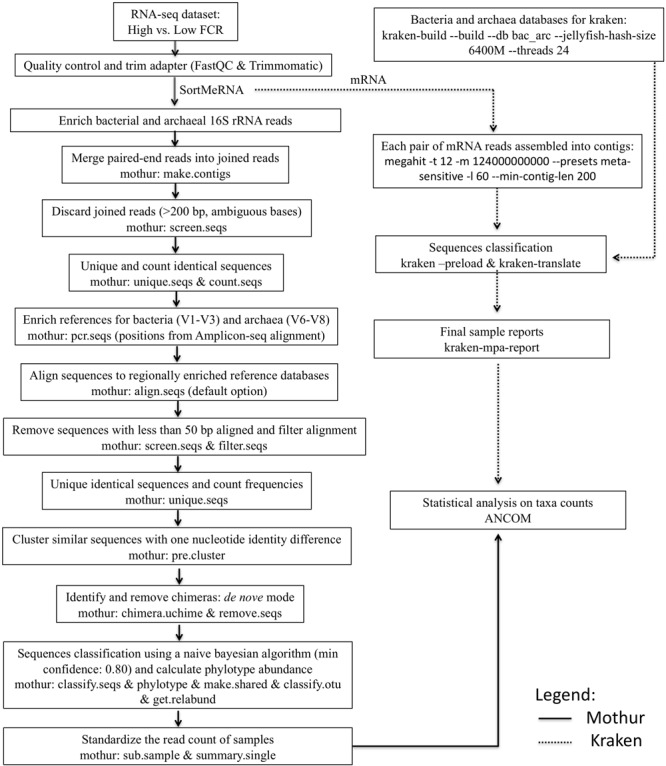
Flow chart of the pipelines (Mothur and Kraken) presenting software parameters used to analyze the rumen microbiota. Part of this figure was adapted from the pipeline published by Li F. et al. (2016).

A flow chart is shown in **Figure 1** to present the software parameters used to obtain the microbial classification from either Mothur (Schloss et al., 2009) or Kraken (Wood and Salzberg, 2014) taxonomic assignment strategies. In the pre-processing steps, all fastq-formatted sequences were firstly uploaded into FastQC^1^ for quality control and removal of ambiguous sequences, and then the software Trimmomatic (version 0.32; Bolger et al., 2014) was used to trim residual artificial sequences, cut bases with quality scores below 20, and remove reads shorter than 50 bp (Li F. et al., 2016). After pre-processing, SortMeRNA (version 1.9; Kopylova et al., 2012) was used to sort the filtered reads into fragments of 16S rRNA (for taxonomic identification using Mothur) based on the rRNA reference databases SILVA_SSU (release 119; Quast et al., 2013) and mRNA (for microbial classification using Kraken). In the pipeline developed by Li F. et al. (2016), sorted paired-end reads belonging to bacterial and archaeal 16S rRNA were joined to increase the read length by combining the forward and reverse sequences. After the 16S rRNA sequences were enriched, downstream analyses were performed using Mothur (version 1.31.2; Schloss et al., 2009) as described by Kozich et al. (2013) (**Figure 1**). For taxonomic classification, bacterial and archaeal 16S rRNA sequences were aligned with the V1–V3 region-enriched Greengenes database (DeSantis et al., 2006) and the V6–V8 region-enriched rumen-specific archaea database (Janssen and Kirs, 2008, which was updated from Kittelmann et al., 2013), respectively. *De novo* chimera detection was then conducted using UCHIME (Edgar et al., 2011), and non-chimeric sequences were taxonomically assessed using a naive Bayesian method (Wang et al., 2007). The pipeline developed by Li F. et al. (2016) will be referred as Mothur through the rest of the paper.

As for the Kraken pipeline (Wood and Salzberg, 2014), newly developed *Perl* scripts were used to retrieve all complete genomes of bacteria (5,294) and archaea (209) from NCBI (RefSeq) (May 2016), to build a Kraken standard database (June 2016) based on their annotations at the lowest taxonomic level (**Figure 1**). Ninety-one complete genomes from organisms isolated from the rumen or from ruminant feces or saliva deposited in the Hungate1000 project were also retrieved from JGI’s IMG database (using NCBI Taxon IDs). After downloading the genomes, the script *kraken-build* (option *–build*) was used to set the lowest common ancestors (LCAs) in a bacteria-archaea joint database (size: 115G; number of sequences mapped to profiles: 10,174; and time for database construction: 6h33m35s). Thereafter, each pair of mRNA sequences was assembled by MEGAHIT (Li et al., 2015), with the resulting contigs (with average extension of 472.31 ± 31.10 bp) being assigned by Kraken (through k-mer discrimination) to the LCA in the customized standard database for microbial classification (**Figure 1**). Full taxonomic names associated with each classified sequence (separated from unclassified reads using *kraken* option *–preload*) and standard ranks (from domain to species) for each taxon were provided by *kraken-translate* and *kraken-mpa-report* (**Figure 1**).

### Statistical Analysis

In this study, a phylotype was considered as classified by both methods if it had at least one count detected in the 12 samples. For comparisons between H-FCR and L-FCR groups, we investigated only bacterial and archaeal profiles with a relative abundance > 0.1% prevalent in at least three samples (3 out 6) to avoid sparsely observed counts, which tend to introduce noise in the analysis (Chen and Li, 2013). The ANCOM procedure (Mandal et al., 2015), which uses an alternative normalization approach called Aitchison’s log-ratio transformation (Aitchison, 1982), was then used to normalize the sequence data and to compare the normalized log ratio of the abundance of each taxon to the abundance of all remaining taxa (Weiss et al., 2017). To deal with zero counts in the datasets, ANCOM used an arbitrary pseudo count value of 0.001 (Mandal et al., 2015). Thereafter, Wilcoxon rank sum tests were calculated on each log ratio to find differences between feed efficiency groups (H-FCR vs. L-FCR) as provided by each classification method (Mothur or Kraken) (**Figure 1**). The *p*-value of each test were adjusted into false discovery rate (FDR) using the Benjamini-Hochberg algorithm (Benjamini and Hochberg, 1995), and a threshold of FDR lower than 0.15 (Korpela et al., 2016) was applied to determine the significance due to the small sample size of this study. Correlation circle plots and relevance networks for core bacterial genera and archaeal species (with a relative abundance > 0.1% detected in all rumen samples; Li and Guan, 2017) were generated from the output of regularized canonical correlation (rCC) analysis as implemented in the R package *mixOmics* (Gonzalez et al., 2008) and Cytoscape 3.4.0 (Shannon et al., 2003). Before running rCC analysis, the data was normalized by total sum scaling (TSS) (dividing each taxon count by the total number of counts in each individual sample to account for uneven sequencing depths across samples) and then transformed by centered log ratio to project the data from a simplex to a Euclidian space (Aitchison, 1982; Mandal et al., 2015; Cao et al., 2016). Then, estimation of regularization parameters (λ1 and λ2) and canonical correlations were calculated using the cross-validation procedure (Gonzalez et al., 2008). Finally, alpha-diversity indexes were calculated using the R package *vegan* (as provided by each classification method) and compared between FCR groups (H-FCR vs. L-FCR) using paired Wilcoxon signed rank test. All statistical procedures were performed using R 3.3.2 (R Core Team, 2016).

### Data Submission

The datasets analyzed in this study were submitted to NCBI Sequence Read Archive (SRA) under the accession number PRJNA403833.

## Results

### Taxonomic Distribution of the Microbial Profiles Performed by Mothur or Kraken

In this study, two bioinformatics approaches, Kraken and a Mothur-based pipeline developed in-house by Li F. et al. (2016), were used to obtain taxonomic classifications (bacteria and archaea) of the ruminal microbiota in bulls exhibiting different (*P* < 0.05) feed efficiencies (average FCR for H-FCR group = 7.64 kg dry matter intake (DMI)/kg gain; average FCR for L-FCR group = 5.71 kg DMI/kg gain; *P* = 0.008). Taking into consideration the total number of microbial taxa in the samples, Kraken identified a higher number of bacterial and archaeal phylotypes at all taxonomic ranks than Mothur (**Table 1**). At the phylum level, the results of bacterial profiles revealed a similar taxa distribution of the most abundant taxa classified by both methods (**Tables 1, 2**), with Bacteroidetes, Firmicutes, and Proteobacteria being highly abundant and accounting for approximately 80% of the total bacterial community. However, Spirochaetes (4.9%) were the fourth-most abundant taxon identified by Kraken, followed by Verrucomicrobia (2.3%), Actinobacteria (2.1%), Tenericutes (1.9%), and Fibrobacteres (1.2%). In contrast, Fibrobacteres (3.4%) was found to be the fourth-most abundant taxon detected by Mothur, followed by Spirochaetes (2.2%), Verrucomicrobia (1.7%), Tenericutes (0.8%), and Cyanobacteria (0.6%). Although there was some congruency (69 commonly detected taxa) at the most resolvable level (up to genus) of bacteria in between the two pipelines, an additional 159 genera were exclusively identified by Kraken. Genera such as *Ruminiclostridium, Lachnoclostridium*, and *Acholeplasma* were uniquely identified by Kraken, whereas *Ruminobacter, Coprococcus, YRC22*, and *Oscillospira* were exclusively detected by Mothur. As for the most abundant genera, Kraken revealed *Prevotella* (33.5%), *Treponema* (4.1%), *Ruminoccocus* (4.1%), *Ruminiclostridium* (3.2%), *Bacteroides* (3.0%), *Butyrivibrio* (2.4%) and *Clostridium* (2.2%) at relatively high abundances, while Mothur identified *Prevotella* (22.6%), *Ruminoccocus* (14.6%), *Ruminobacter* (4.9%), *Fibrobacter* (4.3%), *Treponema* (2.4%), and *Butyrivibrio* (1.2%) as more abundant. It is worth noting that although Mothur could theoretically classify sequences at the species level, it was not able to assign bacterial contigs further than the genus level in the current study. Conversely, Kraken detected 423 species (**Tables 1, 2**) such as *Prevotella ruminicola* (27.6%), *Butyrivibrio proteoclasticus* (2.8%), *Treponema succinifaciens* (2.6%), *Ruminiclostridium* sp KB18 (2.2%), and *Fibrobacter succinogenes* (1.8%). A complete list of all bacteria phylotypes (in all taxonomic ranks) classified by Mothur or Kraken is provided in Supplementary Tables 1, 2, respectively. In addition, the direct comparisons of the bacterial taxonomic assignments obtained from both methods across all samples are included in Supplementary Table 3.

**Table 1:** Quantification of taxonomic phylotypes identified by each method.

Phylotypes	Mothur^1^		Kraken^2^	Commonly detected phylotypes (N°)
	Classified (N°)	Unclassified (N°)	Classified (N°)	
**Bacteria**				
Phyla	23	1	26	16
Families	121	66	204	78
Genera	189	135	348	69
Species	–	–	423	0
**Archaea**				
Phyla	1	1	2	1
Families	3	3	7	2
Genera	4	5	8	1
Species	5	6	8	1

*^1^Pipeline to assess the rumen microbiota developed by Li F. et al. (2016) based on Mothur (Schloss et al., 2009). Clustering-first approaches (such as Mothur) allow the discrimination of unclassified reads (Siegwald et al., 2017). ^2^Metagenomic sequence classification method developed by Wood and Salzberg (2014). Unlike clustering-first approaches, assignment-first tools (such as Kraken) do not allow the discrimination of unclassified reads (Siegwald et al., 2017).*

**Table 2:** Differentially abundant bacteria in efficient (low FCR) and inefficient (high FCR) cattle according to the two classification methods^1,2,3^.

Phylotypes	Mothur		Kraken	
	High (%)	Low (%)	High (%)	Low (%)
**Phyla**				
Bacteroidetes	35.0 ± 8.52	43.2 ± 6.98	37.7 ± 9.75	46.1 ± 11.35
Firmicutes	24.8 ± 9.53	19.2 ± 6.71	28.1 ± 7.01	22.6 ± 6.98
Proteobacteria	20.0 ± 4.88	14.6 ± 3.12	15.7 ± 2.59	12.8 ± 2.63
Fibrobacteres	2.50 ± 0.80	4.4 ± 1.28	1.1 ± 0.42	1.3 ± 0.31
Spirochaetes	2.0 ± 0.50	2.5 ± 0.33	4.6 ± 0.60	5.1 ± 1.02
Verrucomicrobia	1.4 ± 0.19	2.1 ± 0.82	2.1 ± 0.42	2.4 ± 0.62
**Families**				
Prevotellaceae	18.4 ± 6.73	23.7 ± 6.17	26.9 ± 10.48	35.3 ± 13.63
Ruminococcacea	10.2 ± 4.38	7.73 ± 3.89	8.4 ± 3.01	5.7 ± 3.33
Lachnopiraceae	7.1 ± 3.62	5.5 ± 2.23	7.1 ± 1.65	6.1 ± 1.95
Fibrobacteriacea	2.6 ± 0.83	4.6 ± 1.34	1.8 ± 0.37	1.67 ± 0.51
Spirochaetaceae	1.8 ± 0.56	2.3 ± 0.34	4.8 ± 0.73	5.3 ± 1.21
R4 – 41B	0.02 ± 0.037a	0.13 ± 0.118b	–	–
Actinomycetaceae	–	–	0.1 ± 0.03b	0.2 ± 0.04a
**Genera**				
Prevotella	20.0 ± 8.28	25.2 ± 7.10	28.8 ± 10.75	37.5 ± 14.15
Ruminococcus	5.9 ± 2.88	4.4 ± 2.56	3.9 ± 1.70	2.7 ± 1.83
Fibrobacter	3.2 ± 1.12	5.5 ± 1.56	1.4 ± 0.56	1.6 ± 0.34
Butyrivibrio	1.0 ± 0.19	1.3 ± 0.68	2.2 ± 0.29	2.5 ± 1.31
Xenorhabdus	–	–	0.29 ± 0.246a	0.04 ± 0.036b
**Species**				
*Prevotella ruminicola*	–	–	23.0 ± 9.99	31.6 ± 13.15
*Butyrivibrio proteoclasticus*	–	–	2.6 ± 0.33	2.9 ± 1.44
*Ruminiclostridium* sp *KB18*	–	–	2.8 ± 0.98	1.6 ± 1.14
*Fibrobacter succinogens*	–	–	1.6 ± 0.66	1.8 ± 0.37
*Ruminococcus albus*	–	–	1.7 ± 0.89	1.0 ± 0.77

*^1^Statistical comparisons were obtained by the application of ANCOM (Mandal et al., 2015) on taxa counts determined by Mothur (OUTs) or Kraken (K-mers), and thus estimators are comparable only between High (*n* = 6) and Low (*n* = 6) FCR cattle as provided by each classification method. ^2^*P*-values were obtained using Wilcoxon exact test (calculated on the log-ratio matrix; Mandal et al., 2015), and then adjusted to FDR using Benjamini–Hochberg algorithm (Benjamini and Hochberg, 1995). A threshold of FDR < 0.15 was applied to determine significance. Within a row, means with different superscript are statistically different between High and Low FCR cattle for each method (separately). ^3^Blank spaces indicate that the phylotypes were not detected in the dataset either by Mothur or Kraken.*

In terms of archaea identification, both methods exhibited similar results on the abundance of Methanomassiliicoccaceae (previously referred to as RCC), which comprised more than 65% of the total archaeal families (**Table 3**). However, the two methods generated significantly different archaeal profiles at the species level, with 7 species being exclusively identified by Kraken and 4 taxa being exclusively detected by Mothur (**Tables 1, 3**). Only *Methanobrevibacter ruminantium* was commonly detected by the two methods, being the second-most abundant species classified by Mothur and the seventh-most abundant identified by Kraken. A detailed list of archaeal classification (in all taxonomic ranks) for Mothur or Kraken can be found in the Supplementary Tables 1, 2, respectively, together with the information on the direct comparison of the archaeal taxonomic assignments obtained from both methods across all samples included in Supplementary Table 4.

**Table 3:** Differentially abundant archaea in efficient (low FCR) and inefficient (high FCR) cattle according to the two classification methods^1,2,3^.

Phylotypes	Mothur		Kraken	
	High (%)	Low (%)	High (%)	Low (%)
Families				
RCC and relatives	73.2 ± 3.77	71.9 ± 13.12	–	–
Methanomassiliicoccaceae	–	–	65.5 ± 9.92	67.1 ± 11.29
Methanococcaceae	–	–	13.6 ± 8.96a	4.1 ± 4.85b
Methanobacteriaceae	23.9 ± 4.19	24.8 ± 13.75	6.0 ± 6.42	7.0 ± 7.52
Methanosarcinaceae	0.3 ± 0.35	0.6 ± 0.72	5.6 ± 8.51	10.7 ± 5.32
Genera				
Candidatus Methanoplasma	–	–	49.0 ± 13.61	55.4 ± 9.51
Candidatus Methanomethylophilus	–	–	19.0 ± 7.92	12.8 ± 6.78
Methanosarcina	–	–	5.1 ± 9.20	11.0 ± 5.81
Methanobrevibacter	21.8 ± 3.85	21.8 ± 10.53	4.7 ± 5.01	5.3 ± 5.35
Methanosphaera	0.8 ± 0.47	1.2 ± 1.57	–	–
Methanimicrococcus	0.3 ± 0.33	0.6 ± 0.70	–	–
Species				
*Candidatus Methanoplasma termitum*	–	–	51.0 ± 14.27	62.8 ± 11.23
*Candidatus Methanomethylophilus alvus*	–	–	19.7 ± 8.28	14.6 ± 8.09
*Methanobrevibacter gottschalkii and relatives*	14.8 ± 3.60	14.7 ± 6.11	–	–
*Methanobrevibacter ruminantium*	3.8 ± 1.70	4.0 ± 4.19	2.4 ± 4.05	1.2 ± 3.14
*Methanobrevibacter wolinii and relatives*	0.1 ± 0.14	0.2 ± 0.32	–	–
*Methanobrevibacter woesei*	0.1 ± 0.10	0.05 ± 0.06	–	–
*Methanobrevibacter smithii*	0.03 ± 0.07	0.10 ± 0.14	–	–

*^1^Statistical comparisons were obtained by the application of ANCOM (Mandal et al., 2015) on taxa counts determined by Mothur (OUTs) or Kraken (K-mer), and thus estimators are comparable only between High (*n* = 6) and Low (*n* = 6) FCR cattle as provided by each classification method. ^2^*P*-values were obtained using Wilcoxon exact test (calculated on the log-ratio matrix; Mandal et al., 2015), and then adjusted to FDR using Benjamini–Hochberg algorithm (Benjamini and Hochberg, 1995). A threshold of FDR < 0.15 was applied to determine significance. Within a row, means with different superscript are statistically different only between High and Low FCR cattle for each method (separately). ^3^Blank spaces indicate that the phylotypes were not detected in the dataset either by Mothur or Kraken.*

### Differences in Relative Abundances of Taxa in H- vs. L-FCR Rumen Samples

To evaluate how the above two approaches affect the biological interpretation of bacteria and archaea diversity and community structure, comparisons of rumen microbiota between H- and L-FCR cattle were performed. Differences in microbial abundance between H- and L-FCR datasets were found to be minimal (making up less than 1% of the total microbial community), regardless of the classification method (**Tables 2, 3**). In this regard, only the family R4-41B (exclusively detected by Mothur) were more (FDR < 0.15) abundant in the rumen of L-FCR bulls, while the family Actinomycetaceae was more (FDR < 0.15) abundant in L-FCR samples classified by Kraken (**Table 2**). Methanococcaceae and *Xenorhabdus* exhibited a higher (FDR < 0.15) abundance in the rumen of H-FCR bulls when sequences were exclusively classified by Kraken (**Tables 2, 3**).

In addition, alpha-diversity indexes of bacteria (genus level) and archaea (species level) were compared between H- and L-FCR groups to determine how the two pipelines differed in microbial biodiversity estimates. Shannon, Inverse Simpson and Simpson (with rarefy) indexes were higher (*P* < 0.05, paired Wilcoxon signed rank test) in H-FCR than in L-FCR bulls as shown by both pipelines (**Table 4**). On the other hand, a higher (*P* < 0.05, paired Wilcoxon signed rank test) archaeal diversity in the H-FCR group was observed only by the Kraken pipeline (**Table 4**).

**Table 4:** Comparison of bacterial and archaeal alpha-diversity indexes between efficient (low FCR) and inefficient (high FCR) cattle according to the two microbial classification methods^1^

	Bacteria				Archaea			
Indexes	Mothur		Kraken		Mothur		Kraken	
	High	Low	High	Low	High	Low	High	Low
Number of observed phylotypes	244.1 ± 23.88	239.3 ± 19.98	241.1 ± 28.29	224.5 ± 28.37	8.8 ± 1.33	8.8 ± 0.75	5.0 ± 0.89	4.5 ± 1.05
Shannon^2^	2.78 ± 0.12^a^	2.73 ± 0.14^b^	3.93 ± 0.42^a^	3.51 ± 0.62^b^	0.90 ± 0.08	0.91 ± 0.28	1.27 ± 0.19^a^	1.06 ± 0.25^b^
Inverse Simpson	9.8 ± 1.93^a^	8.7 1.82^b^	12.7 ± 5.75^a^	8.8 ± 5.60^b^	1.74 ± 0.13	1.85 ± 0.61	2.95 ± 0.89^a^	2.30 ± 0.64^b^
Simpson (with rarefy)	0.89 ± 0.03^a^	0.88 ± 0.03^b^	0.90 ± 0.07^a^	0.83 ± 0.11^b^	0.42 ± 0.05	0.42 ± 0.15	0.64 ± 0.04^a^	0.54 ± 0.05^b^

*^1^Within a row, means with different superscript were different at *P* < 0.05. Comparison was conducted using paired Wilcoxon signed rank test for bacteria (genus level) and archaea (species level) separately for High and Low FCR cattle as provided by each classification method, and thus estimators between bacterial and archaeal groups are comparable only within each method and between High and Low FCR animals. ^2^Shannon indices showed in the table are the raw values, and the comparison of Shannon indices between High and Low FCR cattle was based on the exponentially transformed values (Jost, 2007) using paired Wilcoxon signed rank test.*

### Potential Interactions between Bacteria and Archaea Detected by Mothur or Kraken

**FIGURE 2:**
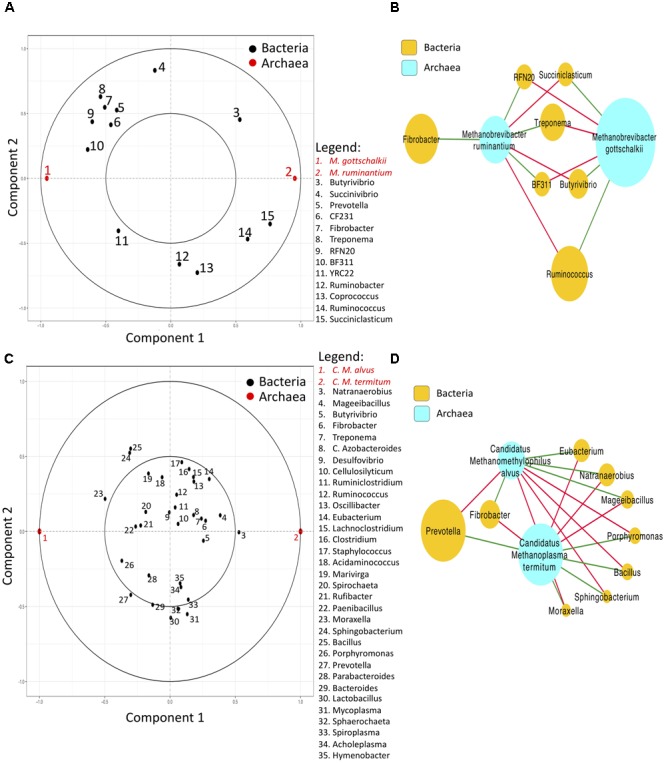
Correlation circle plots and relevance networks generated from the output of regularized canonical correlation (rCC) method (Total Sum Scaling + Centered Log Ratio) applied to rumen bacteria (genera) and archaea (species) classified by Mothur or Kraken. **(A,B)** show the correlation and network plots of the first two rCC components for Mothur. **(C,D)** Represent the correlation and network plots of the first two rCC components for Kraken. In the correlation circle plots, bacteria (X) and archaea (Y) are shown inside a circle of radius 1 centered at the origin, with strongly associated (or correlated) variables being projected in the same direction from the origin. The greater the distance from the origin indicates stronger association. Two circumferences of radius 1 and 0.5 are plotted to reveal the correlation structure of the variables (Gonzalez et al., 2008). In the relevance networks, red and green edges indicate positive and negative correlations respectively, and the sizes of the nodes indicate the mean average abundance. Only bacterial genera and archaeal species with a relative abundance > 0.1% detected in all rumen samples were included in the rCC analysis (Li and Guan, 2017).

To investigate interactions among different taxa classified by Kraken or Mothur, rCC analysis was implemented to identify relationships *within* and *between* bacteria and archaea communities. Our results revealed that bacteria and archaea interactions were quite contrasting between the two methods, with the microbial groups exhibiting different correlation outcomes as shown in **Figure 2**. *Within* bacterial communities, negative correlations between *Prevotella, Treponema, Fibrobacter* and *Ruminobacter, Butyrivibrio*, and *Ruminoccocus* were observed using the Mothur pipeline (**Figure 2A**), while *Prevotella* and *Bacteroides* were negatively correlated with *Treponema, Fibrobacter* and *Ruminoccocus* when Kraken was used (**Figure 2C**). Associations *within* archaeal species were also different between the two methods, with *Methanobrevibacter gottschalkii* and *Methanobrevibacter ruminantium* being negatively correlated with each other from the Mothur pipeline, and *Candidatus Methanoplasma termitum* and *Candidatus Methanomethylophilus alvus* exhibiting negative correlations with each other in the Kraken pipeline (**Figures 2A,C**). Relevance networks of the associations *between* bacteria and archaea revealed a positive correlation between *Methanobrevibacter ruminantium* and *Fibrobacter, RFN20, Treponema*, and *BF311*, and a positive correlation between *Methanobrevibacter gottschalkii* and *Ruminococcus, Butyrivibrio*, and *Succiniclasticum* based on the microbial classification by Mothur (**Figure 2B**). On the other hand, the positive correlations were detected between *Candidatus Methanoplasma termitum* and *Prevotella, Porphyromonas, Bacillus, Sphingobacterium*, and *Moraxella*, as well as between *Candidatus Methanomethylophilus alvus* and *Fibrobacter, Eubacterium, and Mageeibacillus* in the classification provided by Kraken (**Figure 2D**).

## Discussion

In this study, the comparison of taxonomic outcomes of two pipelines, Mothur (developed by Li F. et al., 2016) and Kraken (developed by Wood and Salzberg, 2014, and adapted to the conditions of this study), was performed to determine which is a better approach in rumen microbial classification when total RNA-seq data were used. The advent of high-throughput sequencing has greatly advanced our knowledge of the ecology and functional capacity of rumen microbes and their role in converting low-quality and unusable feedstuffs into energy sources for host productivity (McCann et al., 2017). As a result, an assiduous effort has been made to unveil the linkage between the rumen microbiota and phenotypic traits of interest such as feed efficiency (Li and Guan, 2017), enzyme discovery (Qi et al., 2011) and methane emissions (Kittelmann et al., 2014; Shi et al., 2014; Kamke et al., 2016). Metagenomic studies have shown that the host may regulate the microbiota and its metabolic activity in relation to feed efficiency (FCR) through host-microbiome cross talk genes such as *TSTA3* (*GDP-L-fucose synthetase*) and *Fucl* (*L-fucose isomerase*), suggesting that the relative abundance of these genes could be used as a predictor for host feed efficiency (Roehe et al., 2016). Although the number of rumen metagenomics and metatranscriptomics studies has grown enormously over the last couple of years (McCann et al., 2017), the functional outcomes and biological interpretation of omics data strongly depend on the computational methods used (Simon and Daniel, 2011; Siegwald et al., 2017). In this study, both Mothur and Kraken pipelines showed the rumen of the bulls to be dominated by *Prevotella, Treponema, Ruminoccocus, Fibrobacter*, and *Butyrivibrio*, which are considered as part of a “core bacterial microbiome” (Henderson et al., 2016). In addition to the mutual “core microbiome” shared by the two pipelines at the genus level, Kraken detected a relatively high abundance of (1) *Prevotella ruminicola* (Supplementary Table 2), which is involved in the ruminal digestion of hemicellulose and pectin (Marounek and Duskova, 1999); (2) *Fibrobacter succinogenes* (Supplementary Table 2), a gram-negative, fiber degrader species (Suen et al., 2011); and (3) non-motile species within the *Ruminoccocus* genus (Supplementary Table 2) that share different niches (La Reau et al., 2016): *R. bicirculans*, which selectively utilizes hemicelluloses but not cellulose or arabinoxylan (Wegmann et al., 2014), and *R. albus*, which is capable of digesting cellulose and xylan (Christopherson et al., 2014).

Interestingly, both methods identified about 1% of Cyanobacteria (Supplementary Tables 1, 2), corroborating the findings of previous studies that have reported low abundances of these oxygenic phototrophic bacteria in the rumen of dairy (Scharen et al., 2017) and beef cattle (Li and Guan, 2017), and of camels (Gharechahi et al., 2015). Cyanobacteria are aerobic bacteria that can perform carbohydrate fermentation in a deficient N_2_ concentration (heterocystous) or in a combination of N_2_ deficiency and anoxic conditions (non-heterocystous) (Nandi and Sengupta, 1998). Although the ruminal environment is widely considered to be anaerobic, significant concentrations of O_2_ (60 and 100 nmol/min per mL) can be detected in the rumen fluid (Newbold et al., 1996), indicating that the presence of Cyanobacteria in the rumen may be related to O_2_ scavenging and sugar fermentation performed under restrict aerobic conditions. It is important to mention that although Cyanobacteria has been widely detected in aqueous and soil environments (Williams et al., 2004; Cruz-Martinez et al., 2009), the identification of this phylum in the mammals’ gut has raised critical questions on what roles these organisms may play in aphotic and anaerobic habitats (Soo et al., 2014) like the rumen. Recent researches have reported that gut Cyanobacteria are highly conserved but their 16S rRNA gene phylogenetic tree differed from the photosynthetic Cyanobacteria, which led to the designation of a new candidate class called Melainabacteria (Soo et al., 2014) whose members are capable of fermenting a range of sugars (e.g., glucose, fructose, sorbitol) into acetate and butyrate in the gut (Di Rienzi et al., 2013). Neither Kraken nor Mothur identified Melainabacteria in the samples, demonstrating that further studies are needed to disentangling its role in the rumen.

However, the two methods (Kraken and Mothur) generated microbial classification at different taxonomic levels for rumen bacteria. To completely understand the function of the rumen microbiota, it is essential to identify organisms at the species level since different species, within the same genus, can have varied functions and niches. The Mothur based method was useful to identify a diverse bacterial microbiota from the RNA-seq datasets, but it was not able to classify any of the bacterial sequences further than the genus level (**Tables 1, 2**). Microbial classification up to the species level is a major challenge for clustering-first approaches based on targeted regional 16S rRNA when short (up to 250 bp) or even longer reads generated from total RNA-seq are used to identify environmental microbes (Xiang et al., 2017). Most existing tools (for bacteria and archaea) lack solid probabilistic-based criteria to evaluate the accuracy of taxonomic assignments to determine the best-matched database hits to distinguish multiple species from the targeted sequence region of the 16S rRNA gene (Xiang et al., 2017). To identify bacteria at the species level, sequencing of full length of 16S rRNA is desired and thus future studies need to increase the sequence length to enhance the resolution for microbial identification. For the Kraken based approach, the reference database was built based on all known microbial genomes and as a result it generated a higher resolution (to the species level) of the rumen microbiota, enabling the program to annotate each microbial sequence to the LCAs (Wood and Salzberg, 2014). In this process, k-mer paths formed by Kraken assign a specific weight to each node (equal to the number of sequences associated with the node’s taxon) while increasing the sensitivity of the species classification even if regions (for example, V3–V5) of the 16S rRNA gene were analyzed (Wood and Salzberg, 2014; Valenzuela-González et al., 2016). Consequently, the generation of chimeric trees using short or long input sequences is improbable with Kraken as unlike other programs (such as Ribosomal Database Project classifier and Mothur), it leaves out specific sequences if there is insufficient evidence for classification and they are designated as unclassified (Valenzuela-González et al., 2016). Therefore, inputting short or long environmental sequences (containing most of the 16S hypervariable regions or mRNA sequences) into Kraken may generate a more representative profile of complex microbiomes (Valenzuela-González et al., 2016) like the rumen. However, the lack of reference genomes for rumen microorganisms also limits Kraken. For example, the classification of *Xenorhabdus* (**Table 2**) and *Xenorhabdus doucetiae* [data not shown; relative abundance (%): H-FCR, 0.1 ± 0.10 found in 6 samples; L-FCR, 0%], a motile, gram-negative soil bacterium usually described as being part of entomopathogenic nematode/bacterium symbiotic complex (Furgani et al., 2008) has not been previously reported in amplicon based sequencing (Li F. et al., 2016) or metagenomic/metatranscriptome sequencing (Li and Guan, 2017) of rumen contents. The classification of this bacterial species may indicate that Kraken did not properly identify the microbe since the reference genome information was built mostly from all microbial genomes annotated in the NCBI database. However, these organisms may have been actually detected in the rumen since cattle can consume soil, raising the possibility that their detection was transitory.

It is noteworthy that Methanobrevibacter (family Methanobacteriaceae) was identified in both databases (Supplementary Tables 1, 2, and 4). This genus has been reported to be the most abundant archaeal population in the rumen based on DNA datasets (Kittelmann et al., 2013; Henderson et al., 2016), but it had a lower abundance than Methanomassiliicoccaceae at the RNA level in this study. This result is consistent with the research conducted by Li F. et al. (2016), who reported a predominance of Methanomassiliicoccaceae over Methanobrevibacter in RNA-based datasets when compared to DNA Amplicon-seq outcomes, suggesting that Methanomassiliicoccaceae may be more active in the rumen than Methanobacteriaceae. However, further studies are needed to determine whether the differences in abundance between those two archaeal populations have a methodological influence or are controlled by diet, host animal or management strategies. Unlike bacterial classification, Kraken and Mothur generated contrasting results on archaea identification (**Table 3**), which reflects the divergent taxonomic profiles at the species level. For example, certain archaeal genomes, such as *Methanobrevibacter wolinii* and *Methanobrevibacter woesei*, were only found in the rumen-specific archaea database, as the Kraken standard database lacked these complete genomes. However, Kraken was able to detect *Candidatus Methanoplasma termitum* and *Candidatus Methanomethylophilus alvus*, which were not identified by Mothur pipeline. Li Y. et al. (2016) isolated the archaeon ISO4-H5 (member of the order Methanomassiliicoccales) from the sheep rumen and discovered that this archaeal taxon exhibited genome size (1.9 Mb) and GC content (54%) similar to *Candidatus Methanoplasma termitum* (enriched from the termite gut) and *Candidatus Methanomethylophilus alvus* (enriched from human feces). These two species encode pathways required for hydrogen-dependent methylotrophic methanogenesis by reduction of methyl substrates, without the ability to oxidize methyl substrates to carbon dioxide (Li F. et al., 2016). Thus, it is possible that these microbes reside in the rumen. Future analysis with archaeon ISO4-H5 sequences included in the databases of both pipelines as well as its isolation, culture and characterization may provide further evidence of this possibility.

To further verify how these two methods affected data interpretation, the rumen microbiota of H-FCR and L-FCR bulls were compared based on the taxonomic outcomes generated by the two software packages. Both computational pipelines revealed differences in microbial abundance between H- and L-FCR groups at all taxonomic ranks, with Mothur exclusively identifying a higher abundance of poorly characterized bacterial phylotypes (e.g., R4-41B) in L-FCR bulls (**Table 2**). It has been reported that the abundance of R4-41B was negatively correlated with production traits over the first 12 weeks postpartum in dairy cows (Lima et al., 2015), suggesting that it may have undesirable impacts on the function of the rumen microbiome of L-FCR cattle. Although Kraken identified a relatively higher abundance of *Xenorhabdus* in H-FCR bulls (**Table 2**), this result could be erroneous with further validation needed as described above. However, researchers have enumerated and identified a high number (15.7 × 10^4^ Most Probable Number/g) of chlortetracycline resistant Enterobacteriacea in cattle feces that largely consisted of *Xenorhabdus doucetiae* (Watanabe et al., 2016). Since antimicrobial agents (e. g., chlortetracycline) are typically administered subtherapeutically to beef cattle (Inglis et al., 2005), our results suggest that H-FCR animals may be more susceptible to harbor chlortetracycline resistant bacteria than L-FCR animals in the event of a therapeutic administration of this antibiotic. Further investigations aiming to evaluate the effects of antimicrobial agents (e. g., chlortetracycline) on the development of antimicrobial resistance in *Xenorhabdus* recovered from less efficient cattle (H-FCR) are warranted. Kraken also detected a higher (*P* = 0.09) abundance of Methanococcaceae [relative abundance (%): H-FCR, 13.6 ± 8.96; L-FCR, 4.1 ± 4.85] in the rumen of H-FCR bulls, indicating that Methanococcaceae may play a potential role in the linkages between methanogenesis and reduced feed efficiency in cattle. Although RNA-targeted DNA probes and genomic DNA sequencing have revealed a significant population of this archaeal family residing in the rumen (Janssen and Kirs, 2008) and exhibiting a positive correlation with increased forage content in the diet (Pitta et al., 2016), members of this methanogenic archaea family still need to be cultured from the rumen to test our findings.

Finally, our study demonstrated that both pipelines (Mothur and Kraken) were effective in detecting a lower bacterial diversity in efficient (L-FCR) cattle (**Table 4**), corroborating the recent findings by Li and Guan (2017) and (Shabat et al., 2016) that the rumen microbiota of efficient cattle is less complex and more specialized in harvesting energy from the diet through simpler metabolic networks (e.g., acrylate pathway) than inefficient cattle. However, only Kraken identified a significantly lower diversity in the archaeal community in L-FCR bulls, but this result should be carefully interpreted as many archaea phylotypes classified by Kraken are environmental organisms that have not yet been described in the rumen. For example, the methane-producing archaeon *Methanothermococcus okinawensis* (the third-most abundant archaea taxon classified by Kraken, Supplementary Table 2) was first isolated from a deep-sea hydrothermal vent system (Takai et al., 2002), *Picrophilus torridus* and *Acidilobus saccharovorans* (the fourth and fifth-most abundant archaea taxa detected by Kraken, Supplementary Table 2) were isolated from a dry solfataric field (Fütterer et al., 2004) and a terrestrial acidic hot spring (Mardanov et al., 2010), respectively. Thus, it is worth mentioning that, in spite of the Kraken’s promising results, this pipeline is severely limited when studying a microbiome that is not well described in its standard database (like the rumen), indicating that Mothur (using a specific archaea database described by Li F. et al., 2016) could be more suited for identifying archaeal taxonomic profiles.

## Conclusion

The current study is the first to compare the molecular-phylogenetic outcomes of Mothur and Kraken using transcriptomic sequence data (∼140 bp in length) of rumen samples. The Kraken pipeline has been adapted to include reference genomes for rumen specific organisms, which has led to the identification of rumen bacteria at species level and more bacterial phylotypes. However, the results of the archaeal classification as well as some of the bacterial species identified by Kraken should be carefully interpreted as many detected phylotypes have not yet been described in the rumen, highlighting the importance of strengthening the Kraken database through the inclusion of more genomes annotated by single cell sequencing of rumen cultures/isolates to enable a more accurate classification. As to the future directions, we plan to include new sequenced genomes (410 draft bacterial and archaeal genomes) by Hungate1000 project (JGI database) into Kraken standard database and the recently developed Rumen and Intestinal Methanogen Database (Seedorf et al., 2014) for further analysis, with the goal of improving the accuracy of the results. We also propose the configuration of a joint pipeline using both Kraken and Mothur simultaneously to improve the resolution of taxonomic profiling of the rumen microbiome. This joint pipeline will produce a final rumen microbial profile obtained from the combination of multiple results generated from different bioinformatics tools as outlined by Piro et al. (2017), who published a computational method called *MetaMeta* that executes and integrates results from six metagenomic analysis tools (CLARK – Ounit et al., 2015; DUDes – Piro et al., 2016; GOTTCHA – Freitas et al., 2015; KRAKEN – Wood and Salzberg, 2014; KAIJU – Menzel et al., 2016; and mOTUs – Sunagawa et al., 2013). If the rumen microbiome datasets are strengthened to the same level as the human databases, the joint pipeline will generate more sensitive and reliable results than those of the best single profile (generated separately by each tool) (Piro et al., 2017). We also believe that a joint pipeline supported by a collection of tools could be useful to control sources of variation present in any metagenomics/metatranscriptomic analysis (e.g., analytical pipelines, related databases and software parameters), which will ultimately lead to standardized results and more reliable biological interpretations. In addition, although Kraken has improved the taxonomic assessment at species level, the high number of unclassified sequences (65%) suggests a need for identifying rumen microbes with a more resolved taxonomic classification. Regardless of the approach we undertake, the only way for improvement is through a continued strengthening of the databases by including additional information of whole genome sequencing of rumen isolates as well as single cell sequencing of unculturable rumen microbes, as the ability to culture rumen microorganisms is still limited.

## Author Contributions

AN and LG conceived and designed the experiment. AN and BG executed Kraken, and FL executed the pipeline based on Mothur. AN analyzed the data and performed the statistical analysis. AN, FL, and BG executed the experiment and wrote the manuscript. LG and TM contributed to the experiment and revised the manuscript.

## Conflict of Interest Statement

The authors declare that the research was conducted in the absence of any commercial or financial relationships that could be construed as a potential conflict of interest.

## Footnotes

1 http://www.bioinformatics.babraham.ac.uk/projects/fastqc/
