# Supplementary material for: Enhancing the Resolution of Rumen Microbial Classification from Metatranscriptomic Data Using Kraken and Mothur
Source: Front Microbiol. 2017 Dec 7;8:2445. doi: 10.3389/fmicb.2017.02445 (PMC5725470; doi:10.3389/fmicb.2017.02445)
Supplement: Supplementary file 5 [file Presentation_1.pdf]

Supplementary Material

Enhancing the resolution of rumen microbial classification from metatranscriptomic data using Kraken and Mothur

Andre L. A. Neves,[1] Fuyong Li[1], Bibaswan Ghoshal[1], Tim McAllister[2] and Le L. Guan[1*]

[1]Department of Agricultural, Food and Nutritional Science, University of Alberta, Edmonton, AB, Canada

[2]Lethbridge Research Center, Agriculture and Agri-Food Canada, Lethbridge, AB, Canada

*** Correspondence:**
Dr. Le Luo Guan
lguan@ualberta.ca

1 Supplementary Tables

Here we present four tables containing a complete list of all bacteria and archaea taxa (separated by taxonomic ranks) classified by Mothur (Supplementary Table 1) or Kraken (Supplementary Table 2), and a direct comparison of bacteria (Supplementary Table 3) and archaea (Supplementary Table 4) obtained from both methods at the genus level across all samples.

In Supplementary Tables 1 and 2, the results (presented in descending order based on relative abundance, mean and standard deviation, SD) are shown in each dataset as follows: a) Taxa names are in Column 1; and b) IDs of rumen samples collected from high- and low-feed conversion rate bulls (H- and L-FCR, respectively) are shown from Column 2 to Column 17 (H-FCR animals: 17ZD4H, 1ZD4H, 2094ZD4H, 29ZD4H, 77D4H, 8ZD4H; and L-FCR animals: 126D4L, 162ZD4L, 20LZD4L, 2ZD4L, 60ZD4L, 91ZD4L).

In Supplementary Tables 3 (bacteria) and 4 (archaea), the results (relative abundance, mean, and SD) are shown for each genus classified by Mothur and Kraken across all samples. Taxa names are found in Column 1 and IDs of rumen samples collected from H- and L-FCR bulls can be found from columns 2 to 32. We assigned "NA" (Not Available) when a given taxon was not identified either by Mothur or Kraken.

Legend for Supplementary Tables 3 and 4:

■ Mothur

■ Kraken

Files:

Neves et al., Frontiers Microbiology_Supplementary Table 1_Mothur.xlsx

Neves et al., Frontiers Microbiology_Supplementary Table 2_Kraken.xlsx

Neves et al., Frontiers Microbiology_Supplementary Table 3_Bacteria.xlsx

Neves et al., Frontiers Microbiology_Supplementary Table 4_Archaea.xlsx
